# Adsorption of Polyetheramine-230 on Expansive Clay and Structure Properties Investigation

**DOI:** 10.3390/ma17010025

**Published:** 2023-12-20

**Authors:** Yu Qiu, Zheng Lu, Tingzhou Yan, Jian Li, Haixiang Hu, Hailin Yao

**Affiliations:** 1 State Key Laboratory of Geomechanics and Geotechnical Engineering, Institute of Rock and Soil Mechanics, Chinese Academy of Sciences, Wuhan 430071, China; qiuyu181@mails.ucas.ac.cn (Y.Q.);; 2 University of Chinese Academy of Sciences, Beijing 100049, China; 3 Hubei Key Laboratory of Geo-Environmental Engineering, Wuhan 430071, China; 4 Hubei Communications Planning and Design Institute Co., Ltd., Wuhan 430051, China

**Keywords:** polyetheramine, montmorillonite, swelling inhibitor, adsorption, ions, structure

## Abstract

Polyetheramine (PEA) is a swelling inhibitor used to address engineering challenges arising from the interaction between montmorillonite (Mt) and water. This study comprehensively investigates the adsorption characteristics of PEA on three representative expansive clay samples: Na-Mt, Ca-Mt, and engineered expansive soil. Additionally, the desorption of exchangeable ions is examined. The findings reveal that a two-stage adsorption kinetic model and a pseudo-second-order kinetic model can properly describe the adsorption kinetics of PEA on expansive clays. PEA exhibits a strong capacity for ion exchange with sodium ions, while the exchange capacity for calcium ions is limited. Both protonated and non-protonated PEA contribute to rapid adsorption processes. The adsorption isotherms are well-fitted by the Langmuir and Freundlich models, with the Langmuir model being reasonable. At lower equilibrium concentrations, a higher proportion of the adsorption amount is attributed to ion exchange compared to higher equilibrium concentrations. Ion exchange emerges as the primary factor contributing to the adsorption of PEA on Na-Mt, whereas the adsorption of PEA on Ca-Mt and expansive soil is primarily attributed to physical adsorption by non-protonated PEA. X-ray diffraction results reveal significant intercalation effects of PEA as they penetrate the interlayer space and hinder interlayer ion hydration. Fourier transform infrared spectrum results demonstrate that the adsorption of PEA minimally impacts the framework of Mt structural units but primarily reduces the adsorbed water content. Clay-PEA composites exhibit a decreased affinity for water. Zeta potential experiments indicate that the adsorption of PEA significantly diminishes the surface potential of clay-PEA composite particles, effectively inhibiting their hydration dispersion.

## 1. Introduction

The crystal unit of montmorillonite (Mt) is characterized by the arrangement of two tetrahedral sheets sandwiching one octahedral sheet, along with balanced ions [1,2]. Mt, consisting of layered units, has structural negative charges and a substantial content of exchangeable ions [3]. The structural framework of Mt governs its excellent adsorption properties and modifiability, thereby endowing Mt with substantial potential applications in diverse fields, such as drug delivery, pollution containment, and water treatment [4,5,6,7].

Nonetheless, the widespread occurrence of smectite minerals in nature also gives rise to challenges in various domains [8,9]. For instance, bentonite experiences rapid water adsorption and subsequent dispersion of its layered structure under high-water-content conditions, resulting in challenges such as borehole instability in oil drilling [1,8]. As the predominant mineral constituent in expansive soils, Mt demonstrates water adsorption and desorption-induced swelling–shrinkage behavior [10]. The above characteristics can result in substantial deformations and uneven settlements in construction foundations. Therefore, expansive soil poses typical geotechnical challenges in engineering projects.

The swelling behavior of Mt colloids in aqueous environments primarily stems from the inherent negative charge in its crystal structure resulting from isomorphic substitution and the abundance of exchangeable ions, predominantly sodium and calcium [11,12,13]. The formation of electrical double-layer structure and hydration of interlayer ions in water environments contribute to the pronounced swelling capacity of Mt [14,15,16].

Numerous polymers have been introduced into Mt-water systems to address the issues arising from the swelling process of Mt in water-rich environments [1,17,18]. Extensive research has demonstrated that polymers can disrupt the colloidal clay structure and enhance the stability of clay particles in clay–water systems, leading to flocculation [19,20,21]. The functional groups on polymers facilitate effective adsorption on the surfaces of Mt, thereby altering its swelling and dispersion behaviors in aqueous environments. Concurrently, the adsorption of polymer in clay significantly modifies the physicomechanical properties of clay–polymer composites [22]. Over the past few decades, researchers have extensively investigated the properties of clay–polymer composites with the aim of reducing the negative charge on clay particle surfaces, modifying the types of hydrated ions in clay–water systems, or decreasing the number of hydrated ions [1,23].

Compounds and derivatives containing amine groups have received considerable attention for their ability to inhibit clay swelling in water environments. Among various molecular masses, primary amine groups, and repeating structural features (-C-O-C-), polyetheramines (PEAs) have emerged as a prominent research focus in swelling inhibition [21,24]. Moreover, PEA-230, characterized by low environmental toxicity, good durability, excellent water solubility, and relatively small molecular mass, has been studied as a swelling inhibitor to stabilize soil in geotechnical engineering [25,26]. Several studies have investigated the effects of PEA types and concentrations on the swelling index of samples, employing various microscopic tests to elucidate the mechanisms of swelling inhibition and modulating colloidal stability [21,27]. Results show that PEA-230 can inhibit the swelling of Na-Mt and Ca-Mt in the water environment. However, research on the comprehensive interaction of ions exchange between PEA and clay remains limited, especially for Ca-Mt and engineering expansive soil.

This study examines the interactions between PEA and clay using three representative samples: Na-Mt, Ca-Mt, and engineering expansive soil. The investigation primarily focuses on the variations in time, the adsorption amount of PEA, types of exchangeable ions, and exchanged ion quantities during the adsorption process. Additionally, the structures of Na-Mt-PEA and Ca-Mt-PEA composites are characterized. The quantitative results and composite structure are discussed in an integrated manner. This study provides comprehensive research on the adsorption behavior of PEA on typical expansive clays. It can serve as a reference for the subsequent treatment of expansive clay with PEA to inhibit the expansion of various components.

## 2. Materials and Methods

### 2.1. Materials

**Figure 1:**
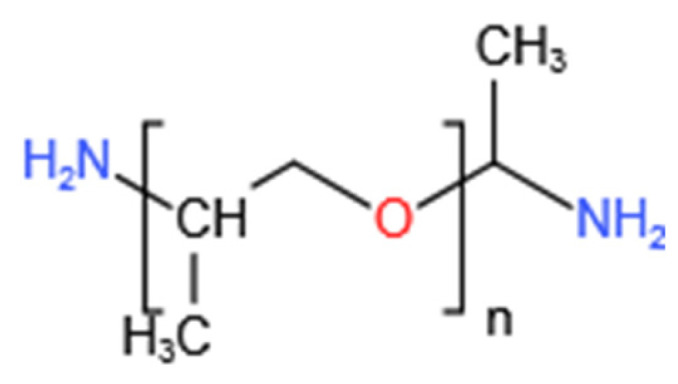
Chemical structure of polyetheramine.

Na-montmorillonite (Na-Mt) was supplied by Beijing Yiwei Specialized Technology Development Co., LTD, Beijing, China, Purity > 98%. Ca-montmorillonite (Ca-Mt) was supplied by Cool Chemistry Technology (Beijing) Co., LTD, Beijing, China, Purity > 90%. Expansive soil (ES) was obtained in Guangxi province, China. The mineral compound includes 11.5% Smectite, 16.1% Illite, 25.7% Kaolinite, 43.8% Quartz, and 2.9% Calcite. The term “expansive soil” is defined in geotechnical engineering. Na-Mt and Ca-Mt are terms for clay minerals. The basic structure of polyetheramine (PEA) is shown in Figure 1. Two terminal amine groups in the molecular chain with a backbone of hydrophobic poly(oxypropylene) for polyetheramine-230 (amine value is 546.02 mg KOH/g) were provided by Aladdin Industrial Corporation. The cationic exchange capacity (CEC) and composition are summarized in Table 1. All clay samples were homogenized and dried to a constant weight before experiments. Deionized water was used throughout all experiments.

### 2.2. Experiments

A series of PEA solutions were prepared, namely, 0, 1, 2, 4, 6, 8, 10, 20, and 30 g/L. Batch adsorption measurements were conducted according to the standard of GB/T2185 with reasonable ratio of adsorbate to adsorbent in polypropylene centrifuge tubes [28]. Mt samples were dispersed in different solutions to prepare 5 g/L suspensions to ensure thorough dispersion. ES samples were dispersed to prepare 200 g/L suspensions according to ratio of macro anti-swelling tests [26]. Adsorption kinetic experiments were performed to evaluate the process of the ion exchange amounts and the adsorbed amount of PEA on the clay. The initial solution concentration was set as 2 g/L PEA, and the equilibration time varied from 0 to 48 h in a thermostatic water oscillator at 150 rpm and at a temperature of 25 °C. The adsorbed amount of samples (48 h) and the corresponding equilibrium concentration were calculated to analyze the adsorption isotherm. The centrifuged period was included in the equilibration time. Na-Mt samples needed to be centrifuged for 3 min at 10,000 rpm, and the supernatant was filtered through a 0.45 μm nylon66 filter using a 10 mL syringe. Ca-Mt and ES samples were filtered without a centrifugation process. The filtrate obtained after filtration, corresponding to different PEA and ion concentrations, was determined by TOC-V_CPN_ Total Organic Carbon Analyzer and Agilent ICP-OES 730. The adsorbed amount of PEA and the exchange amounts of inorganic ions were calculated by determining the difference between the initial concentration and the concentration (filtrate). 

Kinetics models of adsorption processes are described as follows [29,30]:

(1) $$\mathrm{Two-stage}\;\mathrm{sorption}:\;\frac{C_{t}}{C_{0}}=F_{r}e^{-k_{r}t}+(1-F_{r})e^{-k_{s}t},$$

(2) $$\mathrm{Pseudo}\;\mathrm{second-order}:\;\frac{t}{Q_{t}}=\frac{1}{k_{2}Q_{e}^{2}}+\frac{t}{Q_{e}}.$$

Adsorption isotherm models of adsorption processes are described as follows [31,32,33]:

(3) $$\mathrm{Langmuir}\;\mathrm{model}:\;Q_{e}=\frac{Q_{m}K_{L}C_{e}}{1+K_{L}C_{e}},$$

(4) $$\mathrm{Freundlich}\;\mathrm{model}:\;Q_{e}=K_{F}C_{e}^{1/n},$$

where *Q_e_* (mg/g) is the amount of PEA adsorbed at equilibrium; *Q_t_* is the amount of PEA adsorbed on the adsorbent at any time (*t*); *C*_0_ (mg/L) is the initial concentration of solution, *C_t_* (mg/L) is the concentration of solution at any time; and *C_e_* (mg/L) is the equilibrium concentration. *F_r_* denotes the proportion of the fast adsorption component; *k_r_* (h^−1^) is the adsorption rate constant for the fast adsorption component; *k_s_* (h^−1^) is the adsorption rate constant for the slow adsorption component; *K_L_* (L mg^−1^) is the Langmuir constant; *Q_m_* (mg/g) is the maximum adsorption capacity; *K_F_* is the Freundlich constant; and *n* is the Freundlich heterogeneity factor. 

### 2.3. Techniques of Analyses

The supernatant was gathered after the centrifugation process of samples (48 h), and the bottom sediment of Na/Ca-Mt in the tube was prepared as wet XRD samples. Moreover, the extra bottom sediment was then dispersed with equal water to the supernatant amount, and the process of centrifugation and dispersion was repeated three times to water-flush the samples. Then, sediments were dried at 80 °C to a constant weight and ground in an agate mortar. Mt-adsorbed PEA (Mt-PEA) were prepared for XRD, FT-IR, and Zeta potential analyses. 

XRD was conducted on the Bruker D8 Advance using the Cu Kα X-ray radiation at 40 kV and 40 mA (λ = 0.15406 nm). The wet and dried PEA-Mt was scanned from 3° to 65° 2*θ* at 2°/min, and the d-value were calculated with the Bragg equation. Malvern Zetasizer Nano ZS90 was used for detecting the potential of Mt in PEA solutions and the Na/Ca-Mt-PEA in the water at 25 °C and pH = 7. FT-IR spectra of dried Mt-PEA hybrids were acquired on Thermo Scientific (Waltham, MA, USA) Nicolet 6700 using the KBr pressing method at a range of 400–4000 cm^−1^.

## 3. Results and Discussion

### 3.1. Kinetics Tests

**Figure 2:**
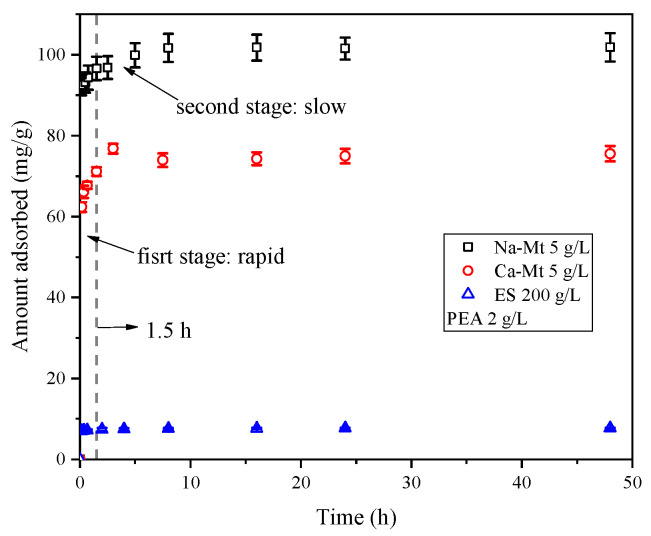
Kinetic adsorption of PEA on Na/Ca-Mt and ES.

As shown in Figure 2, the adsorption process of PEA in Na/Ca-Mt and ES exhibits similar characteristics at 25 °C. Mathematically, the adsorption process can be divided into two stages: rapid and slow. Within the first 15 min, the adsorbed amount increases rapidly, followed by a slower increase. Nearly complete saturated adsorption is reached after 1.5 h. Similar adsorption patterns have been reported in many studies on the adsorption of organic matter on clays [34,35].

Due to the extensive negatively charged sites of the Mt structural units and the rapid expansion of the interlayer space induced by rapid ion hydration, Mt structural units of particle surface can rapidly be dispersed and adsorb a significant number of water molecules. This phenomenon leads to the retention of water molecules on the dispersed surface of units. Water molecules face hindrances in diffusing into the interior of the particles. Consequently, the complete hydration and dispersion of crystal layers within Mt particles in water often require a substantial amount of time. Therefore, the rapid adsorption process of PEA on Mt suggests that PEA rapidly penetrates the liquid film surrounding the Mt particles in the aqueous environment and undergoes adsorption on the particle surface. Furthermore, PEA quickly enters the particle aggregates and undergoes adsorption on both the inner and outer surfaces. Once saturated adsorption is achieved, the entire Mt-PEA-water system reaches equilibrium.

**Figure 3:**
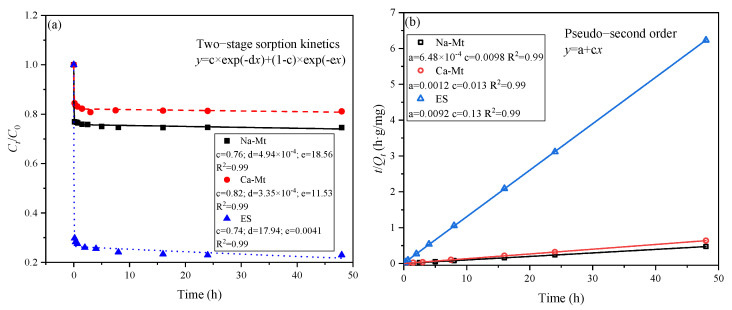
Kinetics fitting curves of PEA adsorption on Na/Ca-Mt and ES: (**a**) two–stage sorption kinetics; (**b**) pseudo–second order.

The two-stage sorption kinetic model assumes that the adsorption process can be divided into two stages, each following a first-order kinetic model [30,36,37]. As depicted in Figure 3, this model effectively captures the adsorption process. The adsorption of PEA by the three types of samples can be simplified into a rapid adsorption stage followed by a slow adsorption stage, with the majority of the adsorbed amount occurring in the first stage. According to the model, it is predicted that 76%, 82%, and 74% of the adsorbed amount takes place in the first stage for the three samples, respectively. The pseudo-second-order kinetic model also exhibits a high degree of fitting to the adsorption process of the three samples [29,38,39]. Consequently, there exist significant limiting factors influencing the adsorption of PEA in the three samples. It can be inferred that the number of active sites on mineral surfaces, the extent of exposure of these sites, and the competitive adsorption of PEA onto these sites control the adsorption process. The slow adsorption process is characterized by the gradual exposure of active sites on mineral surfaces and the competitive adsorption of PEA induced by reduced adsorption sites. According to the pseudo-second-order kinetic model, the equilibrium adsorption capacities of Na/Ca-Mt and ES are predicted to be 102.04, 76.92, and 7.69 mg/g, respectively.

**Figure 4:**
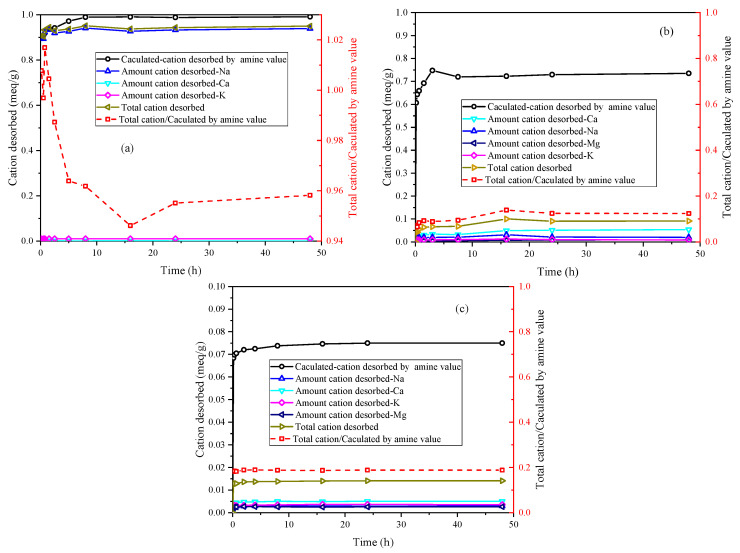
Comparison of calculated-cation desorbed by amine value and amount cation desorbed in the kinetics: (**a**) Na-Mt; (**b**) Ca-Mt; (**c**) ES.

Based on the amine value of PEA and assuming a one-to-one exchange between primary amine groups and unit charge, the calculated-cation desorbed by amine value can be calculated from the adsorbed amount of PEA. The desorption of inorganic cations corresponds to the adsorption of protonated PEA on the samples. Figure 4 illustrates the ion desorption process of PEA in the three samples. The sodium ion is the primary exchangeable ion in Na-Mt, while calcium and potassium ions have relatively low concentrations. The ion desorption process primarily exists in the rapid adsorption stage of PEA, which closely corresponds to the major desorption stage of sodium ions. Consequently, the adsorption of PEA leads to the desorption of inorganic cations. With the desorption of a large number of sodium ions, primary amine groups hydrate to form positively charged groups in the aqueous solution to maintain charge balance with the Mt sheets. The adsorption of PEA on Na-Mt is predominantly attributed to protonated PEA. The ratio curve between the total cation desorbed and the calculated cation desorbed by amine value shows that the total cation desorbed is smaller than the calculated cation desorbed by amine value. The ratio value stabilizes at approximately 0.96. Hence, a small fraction of the adsorption of PEA on Na-Mt occurs through the adsorption of unprotonated PEA rather than ion exchange. The ion desorption process in Ca-Mt aligns well with the rapid adsorption process of PEA. However, a significant discrepancy exists between the total cation desorbed and the adsorption amount of PEA in Ca-Mt. The total cation desorbed is much smaller than the calculated cation desorbed by amine value, and the ratio stabilizes at 0.16. Based on the cation exchange capacity (CEC) and the oxide composition of Ca-Mt, only a small fraction of calcium ions is exchanged by PEA. As a result, most PEA adsorption on Ca-Mt occurs without ion exchange. The adsorption of unprotonated PEA on the surface of Mt layers accounts for the majority of the total adsorption. Therefore, it can be inferred that unprotonated PEA molecules extensively coexist with calcium ions between the Mt sheets, controlling interlayer expansion and ion hydration. The experiment results in ES exhibit similarities to Ca-Mt, with calcium ions as the primary exchangeable ions. Based on the mineral composition, it can be inferred that Mt mineral in ES plays a dominant role in the adsorption of PEA. Similarly, the adsorption of protonated PEA in ES only contributes to a small fraction of the total adsorption, approximately 0.19. A significant amount of inorganic cations still exists within the mineral lattice. Consequently, the adsorption of PEA in the clays exhibits two adsorption systems in water: protonated PEA-clay and unprotonated PEA-clay.

### 3.2. Adsorption Isotherms Tests

**Figure 5:**
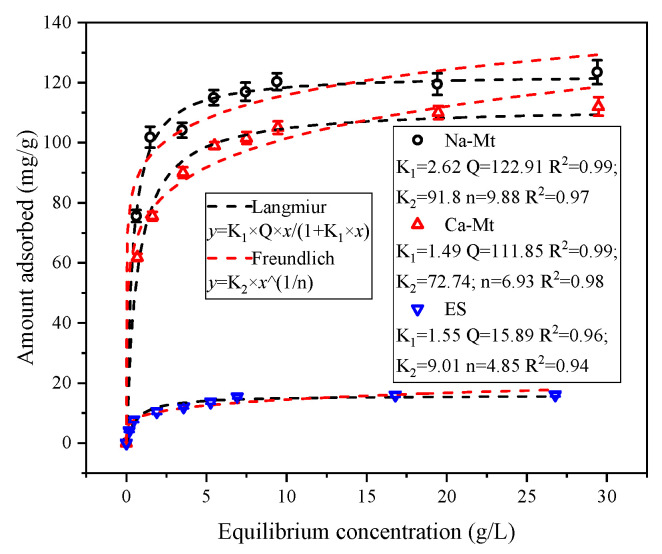
Langmuir and Freundlich fitting curves of the adsorption of PEA on Na/Ca-Mt and ES.

As illustrated in Figure 5, the adsorbed amount of PEA in samples exhibits an increasing trend with the rise in solution equilibrium concentration. Notably, at lower equilibrium concentrations, the adsorbed amount of PEA experiences a rapid escalation. During this phase, the clays disperse rapidly in the solution, exposing interlayer structures and providing a plethora of adsorption sites. PEA molecules are rapidly adsorbed on these sites. The substantial and rapid adsorption of PEA at lower concentrations demonstrates the robust adsorption capacity of clays for PEA. However, as the concentration further increases, the rate of adsorbed amount growth decelerates. The active sites gradually decrease, with the competitive adsorption of PEA molecules limiting the adsorption of PEA on surface sites. Consequently, with the further elevation of the equilibrium concentration of PEA, the adsorption capacity reaches a stable state. Despite a considerable increase in solution concentration, the adsorbed amount of PEA remains unaltered, indicating that the adsorption of PEA on samples has reached saturation.

The adsorption isotherms were fitted using the Langmuir and Freundlich models [31,40]. Both models exhibited a satisfactory correlation with the adsorption isotherms of Na/Ca-Mt while demonstrating a slightly lower correlation with the adsorption isotherms of ES. Although the Freundlich model also effectively depicted the characteristics of the adsorption isotherms, it predicted a further increase in adsorbed amount at high equilibrium concentrations, which contradicts the experimental findings. Consequently, the Langmuir model is deemed more suitable for describing the adsorption of PEA in clays. Consequently, the maximum predicted adsorption capacities of PEA in Na/Ca-Mt and ES are 122.91, 111.85, and 15.89 mg/g, respectively, aligning consistently with the experimental results.

**Figure 6:**
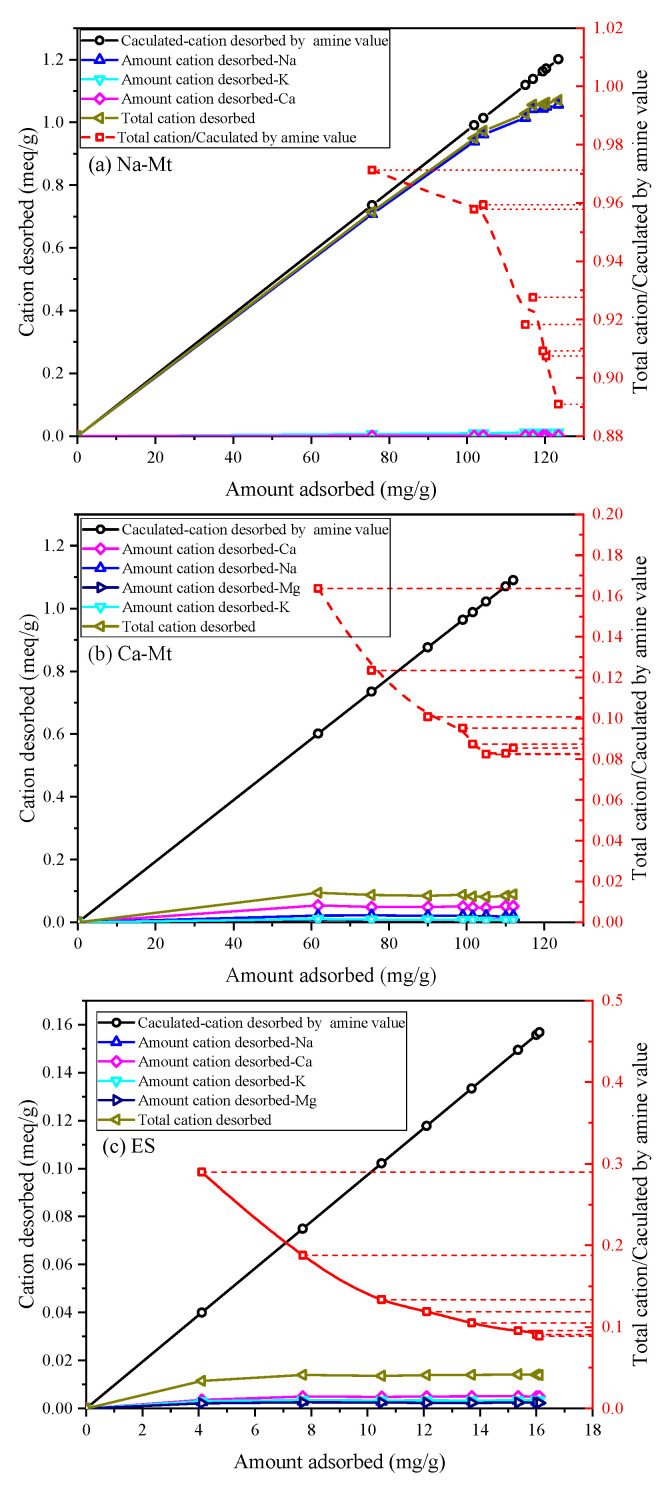
Desorption of ions and total cations accompanying PEA adsorption: (**a**) Na-Mt; (**b**) Ca-Mt; (**c**) ES.

The ion desorption of PEA in the samples is presented in Figure 6. The predominant exchangeable ions are sodium ions in Na-Mt. The total cation desorbed closely aligns with the charge of desorbed sodium ions, showing a positive correlation with the adsorption amount of PEA. Moreover, the isotherm results suggest that higher equilibrium concentrations correspond to larger PEA adsorption amounts. Based on the observed trend of ions desorption, the ratio of total cation desorbed to the calculated cation desorbed by amine value in Na-Mt gradually decreases with increasing equilibrium concentration. Therefore, the proportion of adsorption by non-protonated groups gradually increases as the equilibrium concentration increases.

Further, there are limiting factors to the exchange capacity of sodium ions in Na-Mt, and a complete exchange of all sodium ions from the clay is not achieved. Ion exchange plays a dominant role, but adsorption by non-protonated groups still occurs in the kinetics of the process. Therefore, the adsorption of PEA in Na-Mt is primarily governed by ion exchange. When the maximum exchange capacity for sodium ions is almost reached, the proportion of adsorption by non-protonated groups increases. Furthermore, as the concentration increases, approaching or reaching saturated adsorption on surface sites, the adsorption amount reaches a nearly steady value, along with the desorbed amount of ions.

At lower adsorption amounts of PEA in Ca-Mt, the desorption amount of ions is already close to the maximum value. With an increase in solution concentration, ion exchange is limited, and the proportion of adsorption by non-protonated PEA significantly rises and surpasses the adsorption resulting from ion exchange. Consequently, the primary ion desorption process occurs at low equilibrium concentrations, and increasing the initial solution concentration does not further desorb exchangeable calcium ions. The kinetic results show that ion exchange exhibits a preferential tendency compared to the physical adsorption of non-protonated PEA on the Mt surface. Nevertheless, physical adsorption of non-protonated PEA can also result in a large adsorbed amount on the Mt surface.

Regarding ES, the primary exchangeable ions are predominantly calcium ions, with a lower content of sodium ions. The changing trend of desorbed cations is similar to that observed in Ca-Mt. The primary ion desorption process nearly reaches its maximum value at lower adsorbed amounts. Consequently, as the solution concentration increases, the primary adsorption is mainly due to the physical adsorption of non-protonated PEA. When ES particles come into contact with a PEA solution, PEA rapidly replaces some inorganic cations, resulting in the adsorption of protonated PEA. Once the ion exchange process becomes limited, the physical adsorption of non-protonated PEA further increases the adsorption amount. The greater the proportion of PEA with physical adsorption, the larger the adsorption amount of non-protonated PEA.

### 3.3. X-ray Diffraction (XRD) Analyses

**Figure 7:**
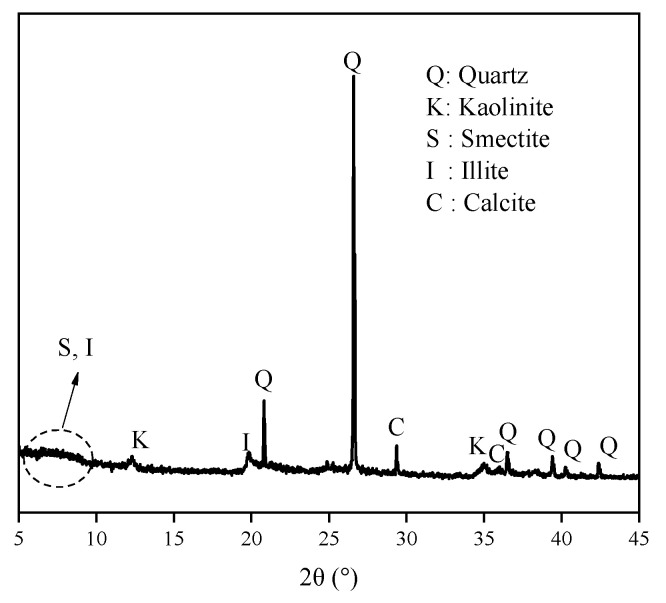
XRD patterns of pristine dried ES.

**Figure 8:**
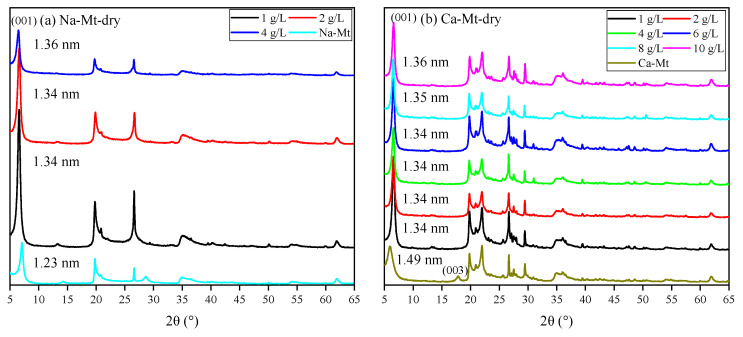
XRD patterns of dried samples with different initial concentrations: (**a**) Na-Mt; (**b**) Ca-Mt.

The XRD spectra of ES are shown in Figure 7. The broad and amorphous reflection in the 5–10° range always revealed mixed-layered minerals of smectite and illite. The reflection position makes it hard to distinguish the displacement. Meanwhile, the components of expansive soil always have obvious regional differences. Thus, discussing PEA on key clay minerals (i.e., Na/Ca-Mt) to induce swelling can directly highlight the influence. The XRD spectra in Figure 8 demonstrate the effect of PEA on the 001 reflection of Mt after completing the adsorption experiments at different initial concentrations. The intercalation effect leads to an increase in the 001 reflection of Na-Mt from the pristine 1.26 nm to around 1.36 nm. On the other hand, the 001 reflection decreases from the pristine 1.49 nm to around 1.36 nm for Ca-Mt. The absence of the 003 reflection in the pristine spectrum of Ca-Mt after the adsorption of PEA indicates a change in the hydration state of ions. Despite higher concentrations of PEA solution resulting in larger adsorption amounts, the change in 001 reflection corresponds to the size of a single primary amine group (0.416 nm). Therefore, the adsorption of PEA between Mt mineral layers occurs only as single-layer adsorption. PEA does not undergo multilayer adsorption on the Mt mineral surface within high-concentration conditions. In the interlayer domain of Na-Mt, protonated PEA molecules and a small amount of sodium ions are predominantly present. In contrast, in the interlayer domain of Ca- Mt, non-protonated PEA coexists with calcium ions. In the dry state of pristine Mt, sodium ions maintain a one-layer water molecule state, while calcium ions maintain a two-layer water molecule state. However, since the size of protonated and non-protonated primary amine groups is larger than that of a one-layer hydrated sodium ion but smaller than that of a two-layer hydrated calcium ion, it can be inferred that the intercalation of PEA molecules affects the hydration state of calcium ions, reducing the size of hydrated calcium ions and consequently decreasing the interlayer space of dry Ca-Mt-PEA.

**Figure 9:**
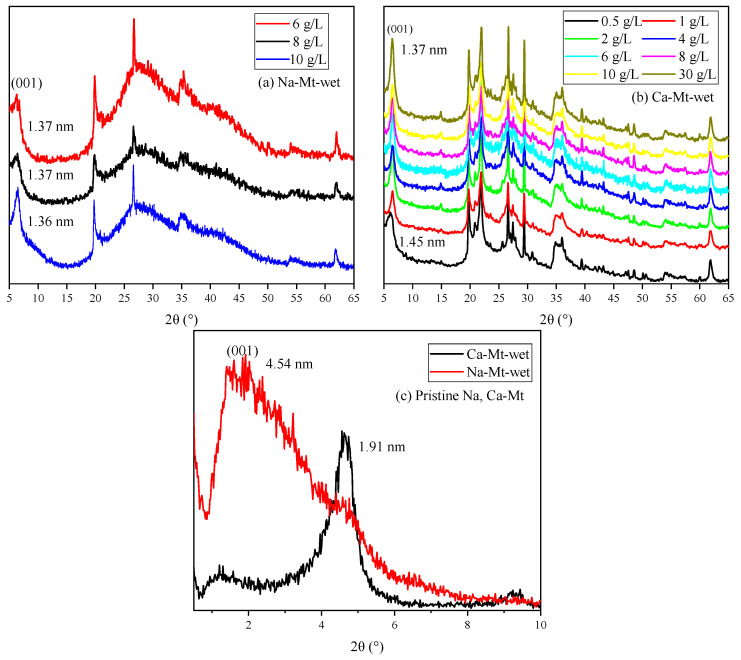
XRD patterns of wet samples with different initial concentrations: (**a**) Na-Mt; (**b**) Ca-Mt; (**c**) pristine Na/Ca-Mt.

The spectra of PEA-Mt and Mt in the wet state are presented in Figure 9, revealing distinct characteristics in the 001 reflections of Na/Ca-Mt. Due to the strong hydration properties of Na-Mt in water, the interlayer space expands further upon the entry of water molecules, reaching a maximum interlayer space beyond the four-layer water molecule state. Obtaining well-defined spectral features for the wet sample of pristine Na-Mt is generally challenging. The displayed spectrum of Na-Mt only indicates the 001 reflection at a specific large interlayer space state. In contrast, the 001 reflection of wet pristine Ca-Mt is measured at 1.91 nm, and it can only be hydrated to the interlayer space corresponding to a less than four-layer water molecule state. This suggests that the layer dissociation of Ca-Mt in water does not undergo further expansion under the four-layer water molecule state. 

The Mt-PEA samples obtained from the adsorption experiment demonstrate a significant inhibitory effect of PEA on the interlayer hydration expansion of Mt in a water-rich environment. However, the effect of dispersion inhibition is insignificant in the Na-Mt sample with lower adsorption amounts of PEA corresponding to a low initial concentration. Additionally, the presence of the polymer and water weakens the intensity and may even mask the peak shape in the spectrum. Only the spectrum of Na-Mt corresponding to a higher initial concentration is obtained, displaying the 001 reflection. The position of the 001 reflection indicates that the interlayer expansion of Na-Mt-PEA is still significantly inhibited in the wet state. The interlayer space remains consistent with the dry sample, measuring 1.36 nm, indicating that the hydration state of interlayer ions does not reach the two-layer water molecule layer. 

Samples of Ca-Mt can be obtained for testing starting from an initial PEA concentration of 0. Importantly, at an initial concentration of 0.5 g/L, the interlayer space is approximately 1.46 nm. Thus, at lower adsorption amounts, the inhibition of interlayer hydration of ions is limited. For the sample with an initial concentration of 1 g/L and an adsorption amount of approximately 63 mg/g, the interlayer space is inhibited to around 1.34–1.36 nm in the corresponding spectrum. With an increase in initial concentration, there is only a minimal shift in the peak position of the 001 reflection. Therefore, the inhibitory effect of PEA on the interlayer expansion of Mt is significant.

### 3.4. Fourier Transform Infrared (FT−IR) Analyses

**Figure 10:**
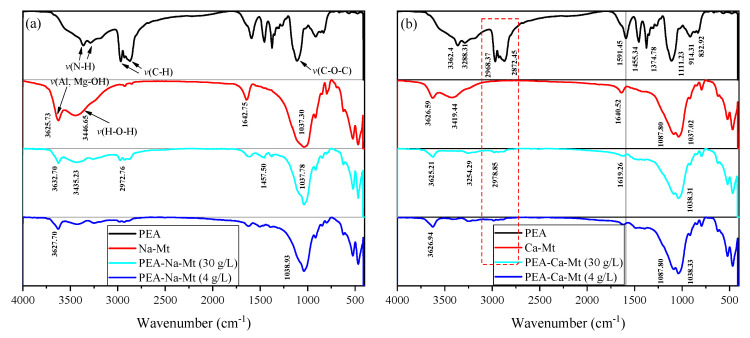
The FT−IR spectra of PEA and Mt-PEA at different concentrations: (**a**) Na-Mt and (**b**) Ca-Mt.

As shown in Figure 10, the spectrum of PEA displays a broad reflection around 3300 cm^−1^, corresponding to the stretching vibration of N-H bonds, while the bending vibration peak is located at approximately 1591 cm^−1^. A characteristic functional group, C-O-C, is also observed at 1111 cm^−1^ [41,42].

The typical adsorption band at 3625 cm^−1^ corresponds to the stretching vibration of O-H bonds in the Al-OH and Mg-OH groups of the layered silicate structure in Na-Mt. An intense and broad absorption band (reflection) is evident around 3446 cm^−1^, representing the stretching vibration of H-O-H bonds in the physically adsorbed water molecules of Mt. Furthermore, the bending vibration of H-O-H bonds occurs at approximately 1642.75 cm^−1^. Similar to the framework structure of Na-Mt, the infrared spectrum of Ca-Mt exhibits a broad absorption band at 3419.44 cm^−1^, which corresponds to the stretching vibration of H-O-H bonds in water molecules. The bending vibration of H-O-H bonds is observed at 1640.52 cm^−1^. The stretching vibration of Al-OH bonds in the framework is observed at 3626.59 cm^−1^. Additionally, the bending vibration peak of H-O-H bonds is generally observed around 1642 cm^−1^. The characteristic bands of Si-O-Si, Si-O-Mg, and Si-O-Al in the primary structure of Mt are characterized primarily in the 1100–400 cm^−1^ range [43,44]. For example, the symmetric and asymmetric vibrations of Si-O-Si in the Mt structure are generally observed at approximately 1037 cm^−1^ and 791 cm^−1^, respectively. 

The adsorbed amount of PEA, corresponding to different initial concentrations, results in the appearance of bands outside the mineral structure itself. Notably, the peaks at 2968 cm^−1^ and 2872 cm^−1^ are associated with the stretching vibration of C-H bonds in methyl and methylene groups. However, the bands corresponding to ether bonds in the primary structure of PEA are obscured by the Si-O-Si band and do not provide sufficient evidence for the adsorption of PEA. The peaks in the primary structure of Mt exhibit no significant changes, indicating that the fundamental framework remains stable before and after PEA adsorption. On the other hand, the shape of the peaks representing water molecules adsorbed in the Mt structure undergoes significant alterations. The distinct stretching vibration peak of H-O-H in pristine Mt is almost absent or indistinguishable, and the bending vibration peak around 1642 cm^−1^ is noticeably weakened. These findings suggest that the adsorption of PEA in Mt samples greatly reduces the water content. The methyl and methylene groups in the molecular structure of PEA are hydrophobic. Therefore, the monolayer adsorption of PEA on the surface of Mt crystal units can create a “hydrophobic film” that impedes the adsorption of water molecules. Furthermore, due to the intercalation effect of PEA, it can be inferred that the PEA molecules in the interlayer domain of the Mt structure unit affect the hydration of inorganic cations.

### 3.5. Zeta Potential (ZP) Tests

**Figure 11:**
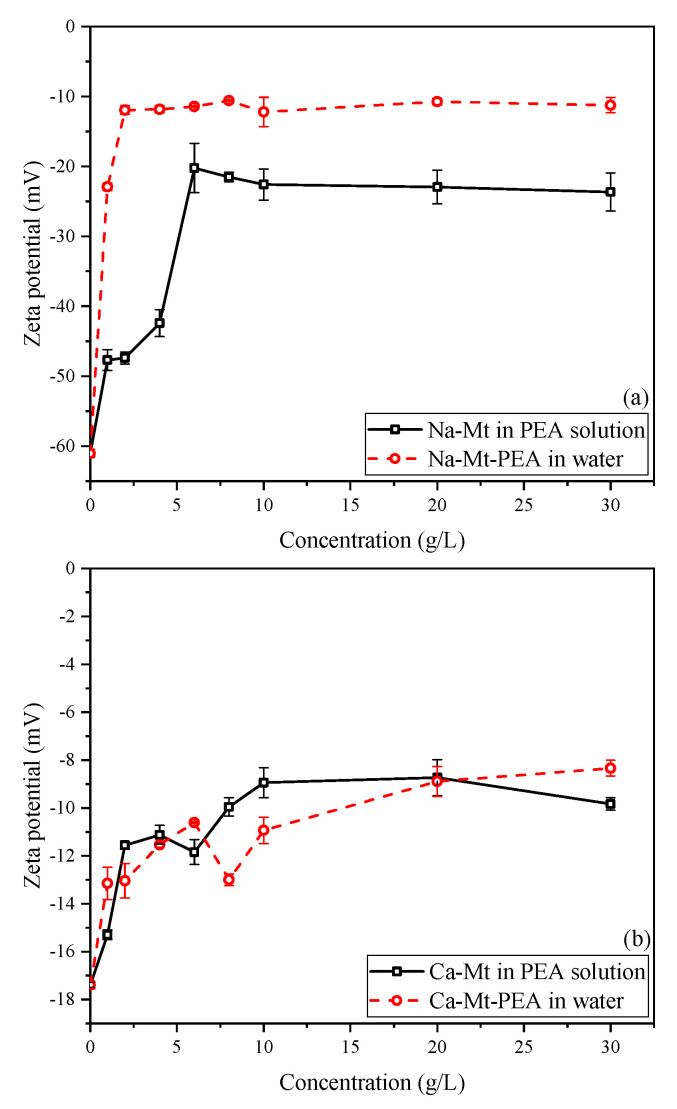
Zeta potential of Mt vs. initial concentration: (**a**) Na-Mt in PEA solutions and Na-Mt-PEA in the water; (**b**) Ca-Mt in PEA solutions and Ca-Mt-PEA in the water.

The zeta potential of particles in a solution system is influenced by various factors, such as background ion strength, particle dispersion, and pH value, which significantly impact the potential of particles in the solution [45,46]. Figure 11a illustrates that the zeta potential of Na-Mt-PEA in water decreases rapidly with increasing PEA concentration and then stabilizes at approximately −10.5 mV as the concentration further increases. Similarly, the zeta potential of Na-Mt samples in PEA solutions rapidly decreases within a low initial concentration range, stabilizing at approximately −22.3 mV. This behavior is similar to previous studies on the zeta potential of Na-Mt particles in solution systems on polymers or large organic molecules [47,48,49]. With concentration increasing, the zeta potential decreases rapidly and then stabilizes to be stable.

The decrease in zeta potential suggests that, with the adsorption of PEA, the potential on the surface of Mt resulting from isomorphic substitution is reduced under the influence of the adsorbed PEA at the double-layer shearing plane formed in the solution. It can be explained as a decrease in the overall negative charge of the Mt particle structure and a reduction in the thickness of the double layer formed by the particles. However, even when the adsorption of PEA on the surface of Na-Mt reaches a steady state, it cannot completely neutralize the overall negative charge of the PEA-Mt structure unit. These findings are consistent with the results reported for polymer-Mt composites regarding zeta potential.

Due to the stronger polarizing ability of divalent calcium ions towards water molecules, interlayer calcium ions can only hydrate up to a distance of approximately four layers of water molecules under water-rich conditions, leading to a lack of dissociation and dispersion of the Mt layers. As shown in Figure 11b, the zeta potential of pristine Ca-Mt particles is approximately −17.3 mV, significantly lower than the −61.2 mV observed for pristine Na-Mt. The zeta potential of Ca-Mt-PEA samples stabilizes at around −9 mV in water. When PEA reaches the maximum adsorption, it also cannot completely neutralize the overall negative charge of the Ca-Mt particles.

### 3.6. Discussion

The research perspective from clay minerals to clayey soil can guide addressing related engineering problems. Discussing the mechanism and macroscopic phenomena associated with PEA has established it as an efficacious material for the remediation of problematic soils. The adsorption of PEA in soil induces diverse changes in the soil’s macro-microstructure, while its modification of the mineral surface effectively inhibits interactions with water. The adsorption of PEA on the soil can result in an increased proportion of larger particles within the soil particle size distribution. Moreover, the enhanced frictional contact among soil particles contributes to greater soil structural stability during soil compaction. Remarkably, the reduced water sensitivity of the PEA-soil hybrids ensures enhanced stability, even in high-water conditions.

Given its good solubility in water, PEA offers versatile applications in various soil treatment methodologies, including infiltration, mixing, and grouting [41,42]. As a result, when confronted with engineering challenges caused by expansive clay, such as problematic borehole walls, roadbeds, and soil slopes, PEA proves to be a suitable soil stabilizer, effectively mitigating these issues.

Moreover, lower levels of PEA adsorption can significantly alter soil properties. As a result, PEA can also be studied to serve as an auxiliary-added material when traditional materials such as lime are added to the soil, providing an additional approach to better addressing soil-related problems. The comprehensive exploration of PEA’s multifaceted applications in soil stabilization showcases its potential as a promising soil stabilizer in the field of soil engineering. 

## 4. Conclusions

**Figure 12:**
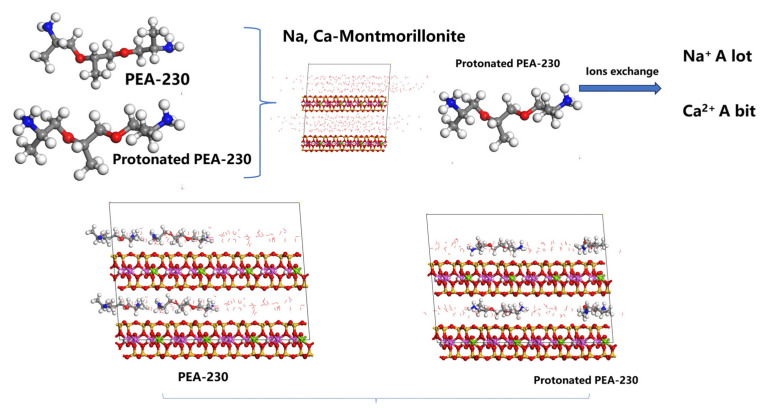
Schematic illustration of the effect of PEA-230 on Na/Ca-Montmorillonite.

As shown in Figure 12, the interaction of PEA and expansive clays is comprehensively investigated. PEA-230 can effectively adsorb on the structural surface of Mt and inhibit the hydration-swelling of the interlayer in the water environment. The effect of PEA on different expansive clays showed potential applications when facing various components of expansive clays with adequate exchangeable Na^+^ or Ca^2+^. The adsorption of PEA on the clays primarily involves rapid ion exchange and physical adsorption of non-protonated PEA. Ion exchange predominantly occurs during the rapid adsorption phase, whereas the physical adsorption process is relatively slower. Under water-rich conditions, PEA rapidly adsorbs on surfaces and enters the interlayer, leading to the exchange of interlayer cations or inhibition of interlayer ion hydration, thereby controlling the interlayer space to approximately 1.36 nm. PEA forms only monolayer adsorption on the surface and significantly reduces the water adsorption capacity of clays.

In the adsorption process on Na-Mt, the adsorption of PEA mainly occurs through ion exchange with sodium ions, although not all exchangeable sodium ions can be exchanged. Adsorption kinetic tests indicate that ion exchange directly contributes to the rapid adsorption of PEA as the adsorption time increases. As the adsorption approaches equilibrium, there is a slight increase in the amount of physical adsorption. With increasing solution concentration, ion exchange remains the primary mechanism for adsorption, albeit with a gradually decreasing proportion in the total adsorption. When reaching the maximum stable adsorbed amount, the adsorption from ion exchange accounts for approximately 0.89 of the total adsorption. The expulsion of a large number of hydrated ions and the inhibition of interlayer hydration expansion reduces the layer dispersion. The adsorption of protonated primary amine groups at negatively charged sites and their inhibitory effect on the hydration layer of inorganic cations lower the zeta potential of Mt particles in aqueous systems.

In the adsorption process on Ca-Mt and ES, ion exchange still occurs during the rapid adsorption phase. After reaching a stable state, the proportion of adsorption from physical adsorption is higher than from ion exchange. Although the proportion of adsorption from ion exchange is relatively low, rapid adsorption can still occur through physical adsorption. With increasing solution concentration, when reaching the maximum stable adsorbed amount, the adsorption from ion exchange of the total adsorption accounts for approximately 0.085 (Ca-Mt) and 0.089 (ES). Therefore, a small amount of hydrated ions is exchanged out of the Mt system. PEA cannot efficiently exchange divalent calcium ions but can still significantly inhibit ion hydration. The adsorption of a small amount of protonated primary amine groups and their inhibition of cation hydration reduces the zeta potential in water environments.

## Figures and Tables

**Table 1:** Basic properties of the clays.

Compound (%)/clay	Na-Mt	Ca-Mt	ES
SiO_2_	65.86	74.91	64.37
Al_2_O_3_	20.79	13.05	20.36
Na_2_O	4.61	0.37	0.15
CaO	0.56	4.37	4.81
MgO	3.66	3.17	1.00
K_2_O	0.35	1.89	1.48
CEC (meq/100 g)	120.8	119.3	61.0
Na^+^	120.2	0.4	0.2
Ca^2+^	0.2	118.4	60.2

## Data Availability

Data are contained within the article.
